# Editorial Notes

**Published:** 1924-07

**Authors:** 


### Editorial Notes.

**The Hospital Problem and the Labour Party.**

THE "Memorandum on Hospital Policy" issued by the Labour Party in 1922, in emphasising the want of organised co-ordination with each other and also with Cottage Hospitals, deficiency in bed accommodation with long lists of patients awaiting admission, and insufficient financial support, was on the whole a fair statement of the Voluntary Hospital position and its shortcomings at that time. But in 1922 the hospitals had barely recovered from the exceptional strain of the war, during which, be it remembered, so much Voluntary Hospital accommodation was allocated for purely military purposes, and when, too, financial stress was almost universal. In the teeth of such odds the Voluntary Hospital System has, nevertheless, displayed such admirable resource and initiative that the gravamen of the criticisms, so true in 1922, has largely disappeared.

We trust that good sense will prevail in maintaining and extending the priceless asset existing in the Voluntary Hospital System. This, however, requires a frank recognition that national needs have outstripped the resources of voluntary effort, that the hospitals are no longer charitable institutions for the necessitous poor alone, but must meet the requirements of an ever-increasing proportion of the community, that the accommodation for in-patients is insufficient, and the equipment and staffing, particularly of the out-patient departments, often leaves much room for improvement, and that voluntary effort must be supplemented by the State, municipality or various public bodies.

At a great price we may exchange the new lamp of a State Medical Service for the old lamp of the Voluntary Hospital System which has so marvellously responded to every hard rub. Maybe it is the success of our Voluntary Hospital System, unsurpassed the world over in record of good service, in striking contrast to the meagre provision by the State for the relief of necessitous patients, which has so awakened our national conscience, that the new-born reforming zeal threatens to eat up the Voluntary Hospitals. Is it possible to breathe into any State service the spirit which called forth and animates the Voluntary Hospital?

In the scheme initiated by Dr. Middleton Martin, with the Bristol Voluntary Hospitals as the chief centre, organised co-operation of the larger hospitals with cottage hospitals in the outlying districts, as well as the institution of post-graduate clinics and arrangements for consultative interchange of opinions between the hospital staffs and practitioners has already been in operation. Again, in our midst we have under the Poor Law Guardians an institution in the Southmead Hospital which is capable of being developed into a well-equipped modern hospital. Here a staff of consultants, comprising physician, surgeon, aurist, physician for children, and a pathologist working in conjunction with the Resident Medical Officers, are doing good work and utilising the resources of the Institution for teaching students.

Thus much that was urged at the Conference held at Caxton Hall in April last under the auspices of the Labour Party not only reflected current opinion but put forward as ideals what is already in process of development.

We already have Municipal Fever Hospitals, Sanatoria for Tuberculosis, Mental Hospital, Clinics under the direction of the Education Committee, etc. Let us welcome and guide the further collaboration of the State, for State aid is not incompatible with voluntary management and voluntary contributions. There is an outstanding example of this in the sanatorium for children suffering from surgical tuberculosis at Zuydcoote in France. This institution was originally founded and maintained by Mons. Vancauwenberg and a group of his friends. The success was so great that in a short time the State decided to recognise the sanatorium. This meant considerable contributions from State and local funds. Mons. Vancauwenberg was asked to remain President, and his friends formed the bulk of the committee of management as formerly. Additional members of committee were nominated by the State and various contributing local authorities, among whom the city of Lille honoured itself by the selection of Professor Calmette. Zuydcoote Sanatorium with its annexe at Berck Plage is now a model of this form of combined management. The contributions of voluntary benefactors, far from having been checked, have been encouraged and stimulated by the fact that the State gave to Mons. Vancauwenberg's experiment the *cachet* of recognition.

We have derived nothing but help and benefit in our Bristol Hospitals from the collaboration of representatives of the workers on our Managing Committee, and we know that from this direction other hospitals have found equal cause for thankfulness. Much may be gained from an organised co-ordination of the Voluntary Hospitals in formulating and presenting the outcome of their experience as a policy for mutual benefit and for the guidance of the State and of municipalities. Certainly we should like to see adequate accommodation for many patients hitherto crowded out of our general hospitals, but even more valuable would be the organised collaboration in representative centres of the Voluntary Hospitals with the Municipal and State services.

Thus in cities like our own we should like to see a committee representing the Voluntary Hospitals, the Municipal medical work and hospitals, the medical work of the Guardians, and of the Education Committee, so that the local effort to provide for all the medical needs of the district may be co-ordinated, and what is undertaken by the State, or by the municipality or by voluntary effort may be the outcome of a policy, not chance. The Voluntary Hospitals have hitherto initiated and led in uplifting the treatment of disease. Shall the State look in vain for their further guidance in developing the wider and more complete developments which they have taught the community to recognise as essential national needs?

**Crippled Children's Claims.**

THE cripple is, in most cases, made, not born. There are approximately 100,000 cripples in this country alone. Here is indeed a problem of national importance. The abolition of conditions which lead to crippledom—overcrowding, malnutrition, lack of sunlight, contact with the tuberculous, milk from cattle of which nearly 20 per cent. are tuberculous—is a matter which can only be solved when the national conscience is properly aroused. Granted, however, that these conditions continue, their evil effects can be minimised by proper organisation.

In Mr. Girdlestone's book[1] the means which can be and have been successfully adopted with this end in view are ably set forth. The first step is diagnosis of the diseases which cause this maiming of our children ere irretrievable damage has been done. Next, treatment both out-patient

[1] "The Care and Cure of Crippled Children." By G. R. Girdlestone, F.R.C.S. Pp. vi., 88. Bristol: John Wright and Sons Ltd. 1924. Price 2s. 6d. net.

and, when necessary hospital, must be available, and must be continued in some cases for a period lasting over many years. During this time there must be facilities for education, and often for specialised vocational instruction to enable the patient to earn a livelihood in after-life. Lastly, there must be efficient after-care and periodical inspection to prevent or to check any tendency to relapse. The general hospital provides next to none of these requirements, since operative treatment by itself is, in the majority of cases, unnecessary, and is all too frequently rendered useless by the lack of after-care. The fresh air and sunlight which have enabled such brilliant cures to be effected in the modern open-air hospital put the city hospital out of court, even were it possible to set aside beds for the years of treatment which many tuberculous cases require.

In this little volume are clearly set forth the steps which have been taken in certain centres to link up the activities of child-welfare associations, boards of health and education, general and special open-air hospitals, local clinics and other organisations. The brilliant results which have followed this co-ordination of effort can only be credited by those who have seen them with their own eyes. Mr. Girdlestone gives a map of England, showing a number of hospital schools for crippled children approved by the Ministry of Health or Board of Education, or both. Save for one little red dot, which represents the 35-bed Orthopædic Hospital at Redland, the whole of the West Country is a blank. Bath has now earned another dot, but there is no city of the size of Bristol which has made less provision for the treatment of its cripples, and it is time that the problem was properly faced.

The book is of absorbing interest. It is well worth reading not only by every medical man, but by all to whom the fate of the cripple makes its appeal, and by the taxpayer whose money at present maintains in the workhouse those who, but for ignorance and neglect, might well be engaged in the happy task of filling up their own income-tax returns.

**The Association of Physicians of Great Britain and Ireland.**

On Friday and Saturday, June 6th and 7th, Bristol entertained the Association of Physicians at its Seventeenth Annual Meeting. The local members, while welcoming the proposal that the Association should meet this year in Bristol, had feared lest the resources of a small medical school might hardly satisfy the needs of the occasion. The event proved that these fears were needless. Indeed, the opinion expressed by many members was that the Association had never enjoyed a better meeting. For this happy result the University is much to be thanked. The Vice-Chancellor received members on the Friday afternoon and took them round the new buildings, the dignity and beauty of which made a deep impression, and also made a delightful speech of welcome at the annual dinner. For the meetings themselves premises which were roomy and yet conveniently compact were found by the courtesy of the departmental chiefs concerned and by the Registrar, who threw himself heartily into the task of preparation. In addition to the morning and afternoon sessions of communications and discussions, there were two demonstrations of clinical cases, a collection of rare old herbals shown by Dr. Newman Neild, including a loan from Dr. J. E. Shaw, series of recent finds exhibited by the Spelæological Society, and a collection of pathological specimens by Drs. Fraser, Hadfield and Todd. It would be difficult to say which of these demonstrations excited the greatest admiration. The President, Dr. George Parker, and the Local Secretary, Dr. J. A. Nixon, are very sincerely to be congratulated on the success with which they organised and conducted these meetings. Among their triumphs must be included the dinner. This was held at the Red Lodge, by the courtesy of the Bristol Savages, whose President, Alderman Fuller Eberle, not only attended the dinner, replying for "The Visitors" to a witty speech by Dr. J. O. Symes, but also exhibited the treasures of the Lodge to his guests.

The Association numbers among its members representatives of nearly every medical school in the British Isles, and we trust that these, with certain visitors from the United States, will treasure with delight their recollection of this visit to Bristol.

**Forbes Fraser Memorial Hospital.**

THE new hospital for paying patients at Combe Park, Bath, opened recently by H.R.H. The Duke of Connaught, owes its inception to the organising skill and enthusiasm of our late colleague, and the decision of the Committee to call it the Forbes Fraser Hospital constitutes a fitting memorial to this distinguished surgeon. It has cost about £40,000, but still requires the further equipment of an X-ray Department to bring it to the degree of completement envisaged by Forbes Fraser.

A fund is to be collected for the purpose of providing this installation, and we cannot doubt that subscriptions will flow in as a tribute to the memory of Forbes Fraser, and ensure that the work to which he set his hand will be rendered complete.

The total cost of the erection and equipment of an X-ray Department is estimated at from £2,500 to £3,000. Donations may be paid direct to The "Forbes Fraser

Memorial Fund," National Provincial Bank, High Street, Bath, or to Mrs. A. L. Hope, *Hon. Sec.*, Eagle House, Bathford, Som.

**Frederick George Farr.**

Our obituary of the late Mr. Frederick George Farr refers to the exceedingly valuable and unstinted work he did for the University Library and the various medical societies, practitioners and students. For this *Journal* during many years he was of the utmost assistance to all concerned in its production, and his knowledge and personality will be very difficult to replace.

We are glad to know that the Committee of the Society have instituted the collection of a fund for the benefit of the late Mr. Farr's widow and young family, and we feel sure that a ready response will render this worthy of the good work so unfortunately ended by his untimely death.

**R. G. Poole Lansdown Memorial Bed.**

In memory of the late Dr. Lansdown his friends and colleagues are collecting a fund, which will be placed at the disposal of the Committee of the Chesterfield Nursing Home, to assist patients requiring operative or other treatment for which a first-class nursing home is needed, but is beyond their means. Chesterfield Home, to the development of which Dr. Lansdown gave unstinted labour, is not conducted for profit, but there is a great want of such means of assisting those above the usual status of patients at general hospitals.

Contributions may be sent to Mr. Cyril Meade-King, Hon. Secretary and Treasurer for the Memorial Fund, c/o Messrs. Meade-King, Orchard Street, Bristol.

